# Structural Characterization and Discrimination of *Morinda officinalis* and Processing *Morinda officinalis* Based on Metabolite Profiling Analysis

**DOI:** 10.3389/fchem.2021.803550

**Published:** 2022-01-21

**Authors:** Liping Kang, Yan Zhang, Li Zhou, Jian Yang, Yali He, Shuai Yang, Gai Li, Qingxiu Hao, Yi Yu, Lanping Guo

**Affiliations:** ^1^ State Key Laboratory of Dao-di Herbs Breeding Base, Joint Laboratory of Infinitus (China) Herbs Quality Research, National Resource Center for Chinese Materia Medica, China Academy of Chinese Medical Sciences, Beijing, China; ^2^ Beijing CACMS-NRC Herbs Testing and Authentication Co., Ltd., Beijing, China; ^3^ North China University of Science and Technology Affiliated Hospital, Tangshan, China; ^4^ Infinitus (China) Company Ltd., Guangzhou, China

**Keywords:** Morindae officinalis radix, processing Morindae officinalis radix, UHPLC-Q-TOF/MS, non-targeted metabolomics, difructose anhydrides, iridoid glycosides

## Abstract

*Morindae officinalis* Radix (MOR) is a famous traditional Chinese medicine (TCM) and functional food material for invigorating kidneys and tonifying yang. Processed *Morindae officinalis* Radix (PMOR) is obtained by steaming MOR. Traditionally, the clinical effects are discrepant between processing and nonprocessing herbal medicines. MOR and PMOR are commonly used in both clinical practice and dietary supplements, and the effect of invigorating kidneys and tonifying yang of PMOR is stronger than MOR. To clarify the overall chemical composition and the difference of MOR and PMOR, a method was developed with an ultrahigh-performance liquid chromatography coupled with quadrupole time-of-flight mass spectrometry (UHPLC-Q-TOF/MS). Among the 110 identified components shared by MOR and PMOR, 55 compounds showed significant differences in contents. Among them, the contents of 29 components, including fructooligosaccharides, monotropein, deacetylasperulosidic acid, geniposide, and anthraquinone glycosides, were higher in MOR than in PMOR; the contents of 26 components, including difructose anhydride sucrose, and iridoid glycoside derivatives, were higher in PMOR than in MOR. Difructose anhydrides and iridoid glycoside derivatives were first discovered in PMOR. These results provided a scientific basis for research on the therapeutic material basis of MOR. It would provide a method for the comparison of processing and nonprocessing in Chinese medicines.

## 1 Introduction

*Morindae officinalis* Radix (MOR), the dried root of *Morinda officinalis* How, is a kind of Chinese herbal medicine widely cultivated in Guangdong, Guangxi, and Fujian provinces of China. MOR is the resource used as both medicine and functional food material with a long history in China. MOR has the effects of invigorating kidneys, tonifying yang, and strengthening bones, and it is used to treat impotence (Zhang, et al., 2018), osteoporosis (He et al., 2019), rheumatoid arthritis (Choi et al., 2005; Zhang et al., 2020), and depression (Cheng et al., 2017). Modern research has reported that MOR has the effects of enhancing immunity, providing antistress and cardiovascular protection, and regulating gut microbiota (Qiu et al., 2016). Processing of TCM is a unique traditional pharmaceutical technique in Chinese medicine. The curative effect of TCM can be improved through processing, the toxic and side effects of the medicine can be eliminated or reduced, the properties and flavor of the medicine can be changed, and new medicinal effects can be produced. Steaming is one of the most common processing methods (Pharmacopoeia of the People’s Republic of China, 2020; Cheng et al., 2019). Processed *Morindae officinalis* Radix (PMOR) is obtained by steaming MOR. Traditionally, it has been thought that the effect of invigorating kidneys by PMOR is stronger than MOR (Cui et al., 2013), but the change in chemical composition of MOR before and after steaming is not clear. The main components of MOR are polysaccharides, oligosaccharides, iridoid glycosides, anthraquinones, etc. (Wang et al., 1992; Hao et al., 2019; Wang et al., 2019). A report showed that MOR contains a large amount of fructooligosaccharide (GFn, *n* ≦ 12) (Hao et al., 2019). The antidepressant effect of MOR may be related to the effect of oligosaccharides on intestinal flora (Burokas et al., 2017). At present, no systematic research report is available in the overall chemical composition and difference between MOR and PMOR. Because of its high resolution and accurate ion mass value, UHPLC-Q-TOF/MS, as a powerful qualitative and quantitative analysis method, has been increasingly used in the analysis of the chemical composition of complex systems, such as botanical medicines (Xu et al., 2018).

To clarify the overall chemical composition and the difference of MOR and PMOR, methods were developed with UHPLC-Q-TOF/MS combined with multivariate statistical analysis methods, such as principal component analysis (PCA) and orthogonal partial least-squares discriminant analysis (OPLS-DA) to systematically study the overall chemical composition in MOR and PMOR, and clarify the similarities and differences in the composition of MOR and PMOR. It was expected to provide a material basis for research on the therapeutics of MOR and PMOR, and provide a potentially useful method for other processed and nonprocessed Chinese medicines.

## 2 Materials and Methods

### 2.1 Chemicals, Reagents, and Herbal Materials

Methanol and acetonitrile (HPLC grade) were purchased from Fisher Scientific Co. (Loughborough, United Kingdom). Ammonia (HPLC grade) was purchased from Merck KGaA (Darmstadt, Germany). Formic acid (HPLC grade) was purchased from Sigma-Aldrich (St. Louis, MO, United States). Deionized water (18.2 MΩ) was further purified using a Milli-Q system (Millipore, MA, United States). Sucrose, 1-kestose (GF2), nystose (GF3), and 1F-fructofuranosylnyslose (GF4) were purchased from the National Institutes for Food and Drug Control, China. GF5, GF6, GF, GF8, and GF9 were purchased from Chengdu Must Bio-Technology Co., Ltd., Sichuan Province, China. Deacetylasperulosidic acid methylester, asperuloside, physcion, 1,8-dihydroxyanthraquinone, 1-hydroxy-2-methylanthraquinone, rhein, emodin, purpuri, rubiadin, shanzhiside methylester, and apigenin were purchased from Beijing Rongcheng Xinde Technology Development Co., Ltd. Monotropein, deacetylasperulosidic acid (DAA), luteolin, cynaroside, alizarin, and aloeemodin were purchased from Shanghai Yuanye Biotechnology Co., Ltd. The purities of reference substances were all HPLC ≥ 98%. GF10 (69.4% GF10, 9.5% GF9, 17.6% GF11, and 2.7% GF12) and GF11 (75.2% GF11, 16.1% GF10, and 4.1% GF12) were purchased from elicityl-oligotech.com (France). The other reagents were obtained in analytical grade.

MOR and PMOR samples were collected from Guangxi, Fujian, and Guangdong provinces as well as the domestic mainstream Chinese herbal medicine market, with a total of 73 batches, including 41 batches of MOR (wood core removed) and 32 batches of PMOR. MOR and PMOR samples were identified as the rhizome of *Morindae officinalis* How by Prof. Jinda Hao (China Academy of Chinese Medical Sciences), and the voucher specimens were deposited in the National Resource Center for Chinese Materia Medica (Beijing).

### 2.2 Sample Preparation

Each sample was freeze dried, crushed into powder with a conventional ball mill (Retsch MM400, GmbH, Haan, Germany), and sifted through a 40-mesh screen. The powder for each sample was weighed at 100 mg (Mettler, Switzerland) and suspended in 2 ml of 70% (v/v) aqueous methanol. Then the extract solution was ultrasonically extracted (KQ-100DE ultrasonic cleaner, Kunshan Ultrasonic Instrument Co., Ltd.) at a 40-kHz frequency for 30 min at room temperature. The extraction was centrifuged at 12,000 rpm for 10 min with a centrifuge (5810R, Eppendorf, Germany). The supernatant was collected and filtered through a 0.22-μm syringe filter (Pall, United States). Each sample solution was stored at room temperature for analysis. The mixed MOR and PMOR extract was the control sample.

### 2.3 Instruments

The UHPLC separation for secondary metabolites was performed using a Waters Acquity UPLC-I-Class system (Waters Corp., Milford, MA, United States) with an Acquity BEH column (100 mm × 2.1 mm, 1.7 μm) for chromatographic separation. The column temperature was set at 40°C, and the flow rate was 500 μl/min. Mobile phase A consisted of a 0.1% formic acid in acetonitrile, and mobile phase B was 0.1% formic acid aqueous solution. The gradient elution program was as follows: 0–0.2 min, 2.0%→2.0% A; 0.2–0.5 min, 2.0%→5.0% A; 0.5–4.5 min, 5.0%→16.0% A; 4.5–10.0 min, 16.0%→36.0% A; 10.0–14.0 min, 36.0%→40.0% A; 14.0–15.5 min, 40.0%→55.0% A; 15.5–21.5 min, 55.0%→63.0% A; 21.5–22.0 min, 63.0%→98.0% A. The sample injection volume was 5.0 μl.

The UHPLC separation for sugars was performed using a Waters Acquity UPLC-I-Class system (Waters Corp., Milford, MA, United States) with an Acquity BEH amide column (100 mm × 2.1 mm, 1.7 μm) for chromatographic separation. The column temperature was set at 40°C, and the flow rate was 200 μl/min. Mobile phase A consisted of a 0.1% ammonium hydroxide in acetonitrile, and mobile phase B was 0.1% ammonium hydroxide aqueous solution. The gradient elution program was as follows: 0–0.5 min, 98% A; 0.5–0.2 min, 98%→89% A; 2.0–2.5 min, 89%→86% A; 2.5–5.5 min, 86%→80% A; 5.5–6.5 min, 80%→73% A; 6.5–9.5 min, 73%→63% A; 9.5–14 min, 50%→50% A; 14–16 min, 50% A; 16.0–16.5 min, 50.0%→98.0% A; 16.6–20.0 min, 98.0% A. The sample injection volume was 1.0 μl.

The TOF MS experiments were performed using a Xevo G2-S Q-TOF MS system (Waters Corp., Milford, MA, United States) in both the ESI (+) and ESI (−) ionization modes. The data acquisition modes were MS^E^ continuum. The source and desolvation temperatures were 100°C and 450°C, respectively, and the desolvation gas flow rate was 900 L/h. The capillary voltages were 0.5 and 2 kV for the ESI (+) and ESI (−) experiments, respectively. The cone voltage was 40 V. The collision energy was set as 6 eV (trap) for the low-energy scans, and as 20–50 eV ramps in high-energy scans. The data acquisition range was 50–2,500 Da. The mass accuracy was maintained using a lock spray with leucine enkephalin (200 pg/μl) as the reference [*m/z* 556.2766 ESI (+) and 554.2620 ESI (–)].

### 2.4 Data Processing and Multivariate Analysis

The instrument was controlled by the Masslynx 4.1 software (Waters Corp., Milford, MA, United States). All the MS^E^ continuum data were processed using the apex peak detection and alignment algorithms in UNIFI 1.8 (Waters Corp., Milford, United States). This processing procedure enables related ions (quasimolecular ion peaks, salt adduct ions, and dehydration fragment ions) to be analyzed as a single entity. Both the TCM library and an inhouse library were employed to characterize the metabolites. All the MS^E^ centroid data were processed using Progenesis QI V2.0 (Waters Corp., Milford, United States). Multiple adduct ions, including [M−H]^−^, [M+HCOO]^−^, [M+Cl]^−^, [2M−H]^−^, [2M+HCOO]^−^, [M−2H]^2−^, [M−H+HCOO]^2−^, and [M−3H]^3−^, were selected or self-edited to remove redundant adduct ion species. The data of apex peak detection and alignment algorithms were processed in Progenesis QI. The intensity of each ion was normalized by the total ion count to generate a marker consisting of *m/z* value, normalized peak area, and the retention time. The normalized peak areas and peak ID (RT and *m/z* pair) were exported to the EZinfo 3.0 software (Waters Corporation, Milford, MA, United States) for multivariate analysis. Analysis methods of PCA and OPLS-DA were used to analyze the main difference components between MOR and PMOR.

## 3 Results and Discussion

### 3.1 Optimization of Operating Conditions

**FIGURE 1:**
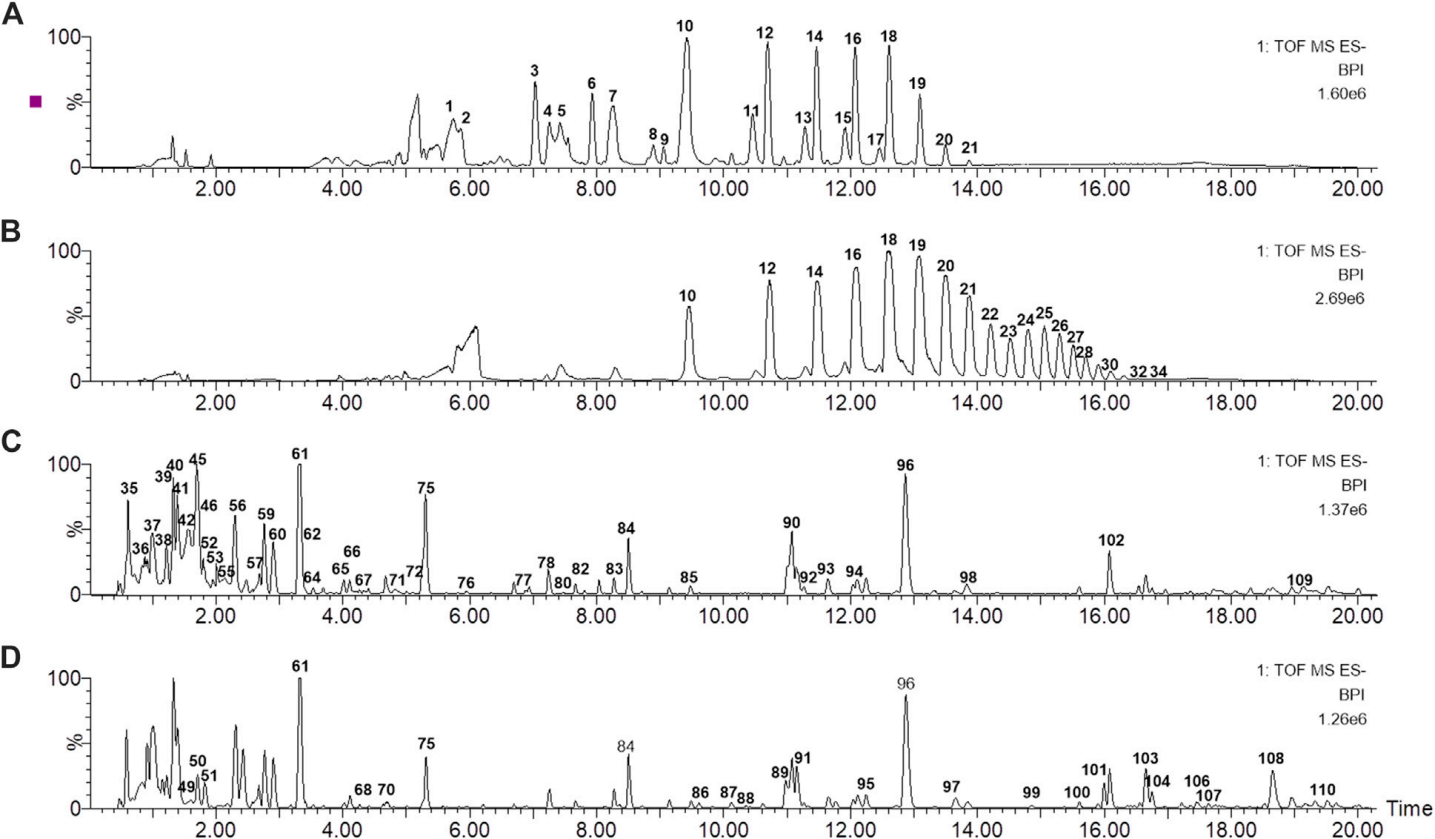
Base peak ion chromatograms of processed *Morindae officinalis* Radix (PMOR) **(A,C)** and *Morindae officinalis* Radix (MOR) **(B,D)** analyzed by ultrahigh-performance liquid chromatography coupled with quadrupole time-of-flight mass spectrometry (UHPLC-Q-TOF MS) in the negative ion mode. **(A,B)** were analyzed with an Acquity BEH amide column; **(C,D)** were analyzed with an Acquity BEH column.

To better characterize the primary and secondary metabolites of MOR and PMOR, the UHPLC-Q-TOF/MS operating conditions were optimized. Base peak ion chromatograms of MOR and PMOR are shown in Figure 1. The Acquity UPLC BEH Amide C18 column could effectively separate fructose, glucose, sucrose, and GF2–GF21 in PMOR (Figure 1A) and MOR (Figure 1B), but it could not well characterize the secondary metabolites in MOR and PMOR. The addition of 0.1% aqueous ammonia helped improve the peak shapes and the resolution. The Acquity UPLC BEH C18 column could effectively separate the components other than sugars in PMOR (Figure 1C) and MOR (Figure 1D). The addition of 0.1% formic acid helped improve the peak shapes and the detection and identification of glycosides. In this study, these two chromatographic separation methods were combined to comprehensively characterize the chemical components of MOR and PMOR, and to find and identify the main compositional differences between these two substances. In the negative ion mode, the responses of sugars and iridoids were better, while in the positive ion mode, the anthraquinone components exhibited rich signals, and so the positive and negative ion modes were selected for UHPLC-Q-TOF/MS data collection.

### 3.2 Analysis of the Main Compositional Differences Between *Morindae officinalis* Radix and Processed *Morindae officinalis* Radix

The results showed that the chemical compositions of MOR and PMOR were significantly different (Figure 1). The chromatographic peaks of sugars with a higher degree of polymerization, especially the oligosaccharides (GFn, *n* > 8), were considerably reduced or almost disappeared, but between 6.8 and 9.2 min, new chromatographic peaks were observed, suggesting that new chemical components were generated in PMOR (Figures 1A,B); In Figures 1C,D, it revealed that a significant difference in the strong polar components, with a retention time of less than 3.0 min, and the chromatograms, after the retention time of 3.0 min, were roughly similar in MOR and PMOR.

**FIGURE 2:**
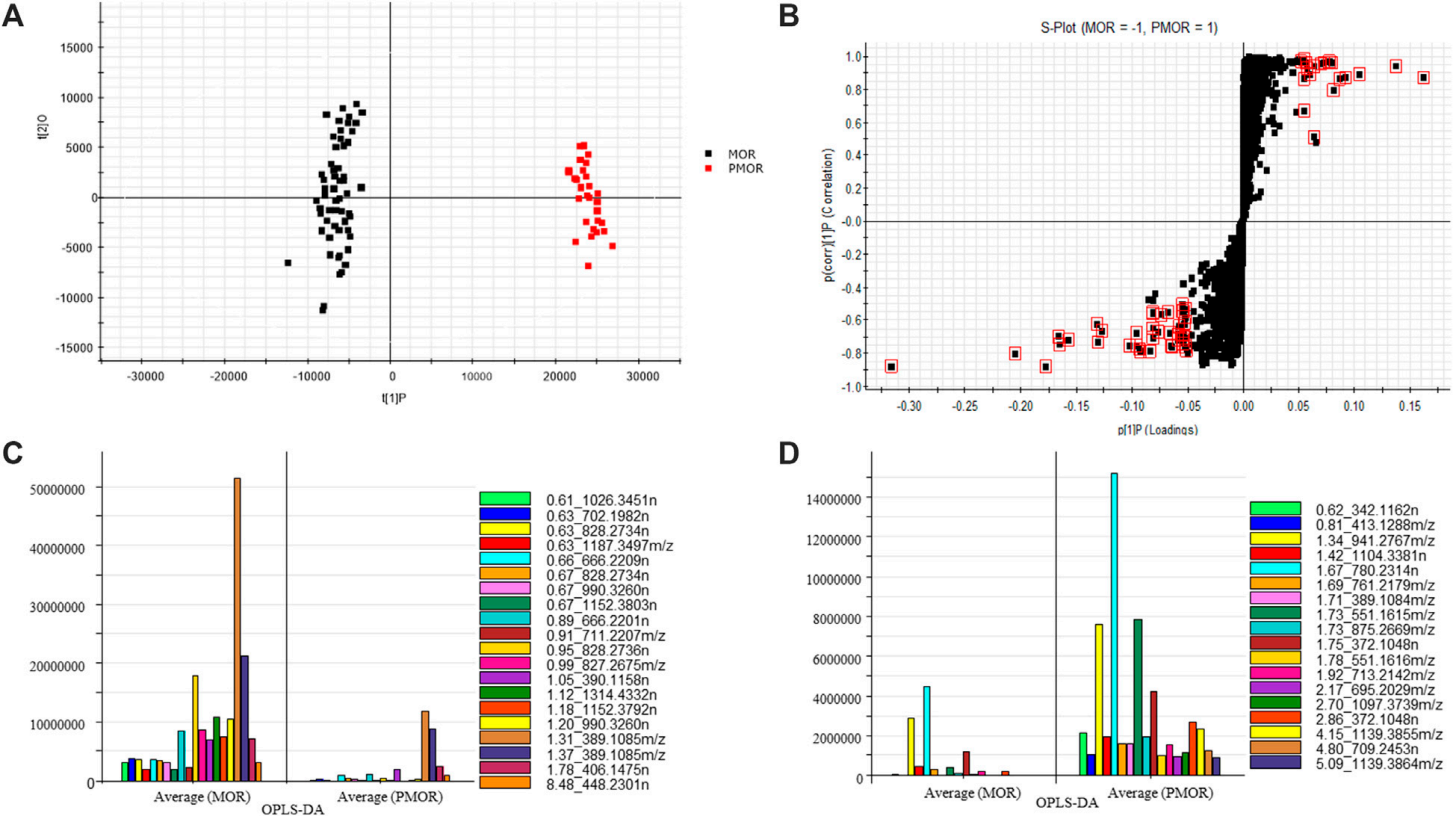
Selected potential chemical markers from MOR and PMOR based on principal component analysis (PCA) and orthogonal partial least-squares discriminant analysis (OPLS-DA) (in the negative ion mode with an Acquity BEH column). **(A)** PCA score plot; **(B)** S-plot, the compounds with large differences in content (which is marked in red squares) between MOR and PMOR; **(C)** the selected chemical markers with content in MOR that is higher than that in PMOR; **(D)** the selected chemical markers with content in PMOR that is higher than that in MOR.

The UHPLC-Q-TOF MS data were processed by Progenesis QI, and the obtained two-dimensional dataset was input into EZinfo software for multivariate statistical analysis. The results of PCA revealed that the samples within each group of MOR and PMOR were clustered into one group (Figure 2A), and the classification between the two groups was obvious, indicating that the chemical compositions of different batches of samples in each group were relatively similar, and the difference in chemical composition between two group samples was large. To find the markers with high contribution to the difference between MOR and PMOR by OPLS-DA, the result is shown in the S-Plot (Figure 2B). In Figure 2B, it showed that each point represented a marker composed of accurate mass and retention time. The farther the point was from the center, the greater the contribution to the difference between the two groups. The value of VIP (variable importance plot) displays the relative influence that each of the predictors (x variables) has on all the responses combined, from the most to the least influential. The data points with scores of VIP ≥5.0 (red data points in Figure 2B) were selected for analysis of the chemical components that differ greatly between the two groups of samples. The retention times, *m/z*, and fold change of these data points are shown in Figures 2C,D. In Figure 2C, it indicates the compounds with a higher content in MOR than in PMOR. In Figure 2D, it shows the compounds with a higher content in PMOR than in MOR. According to the retention time and the mass spectrum information of molecular ion peaks, fragment ion, or neutral loss, it was preliminarily concluded that these components with large differences in content were sugars, iridoid glycosides, and anthraquinone glycosides. By identifying these compounds, the structures of the main different compounds whose content differs the most between MOR and PMOR could be determined (Tables 1, 2).

**TABLE 1:** Chemical components with larger difference in content between the samples of MOR and PMOR (Acquity BEH amide column).

No	*m/z*	RT/min	Formula	Compound	Factor of change	No	*m/z*	RT/min	Formula	Compound	Factor of change
1^a^ ^,^ ^c^	389.1077	5.74	C_16_H_22_O_11_	DDA	4.3	16^a^ ^,^ ^c^	1,475.4782	13.87	C_54_H_92_O_46_	GF_8_	15.9
2^a^ ^,^ ^c^	389.1080	5.84	C_16_H_22_O_11_	Monotropein	2.4	17^a^ ^,^ ^c^	1,637.5297	14.21	C_60_H_102_O_51_	GF_9_	20.1
3^b^ ^,^ ^d^	323.1024	7.03	C_12_H_20_O_10_	Difructose anhydride	>100	18^a^ ^,^ ^c^	1,799.5851	14.52	C_66_H_112_O_56_	GF_10_	21.3
4^b^ ^,^ ^d^	323.0981	7.25	C_12_H_20_O_10_	Difructose anhydride, isomer	>100	19^a^ ^,^ ^c^	1,961.6327	14.80	C_72_H_122_O_61_	GF_11_	34.3
5^b^ ^,^ ^d^	323.0979	7.55	C_12_H_20_O_10_	Difructose anhydride, isomer	>100	20^a^	2,123.6873	15.05	C_78_H_132_O_66_	GF_12_	>100
6^b^ ^,^ ^d^	323.0987	7.93	C_12_H_20_O_10_	Difructose anhydride, isomer	>100	21^a^	2,285.7463	15.30	C_84_H_142_O_71_	GF_13_	>100
7^b^ ^,^ ^d^	323.0980	8.25	C_12_H_20_O_10_	Difructose anhydride, isomer	>100	22^a^	2,447.7971	15.52	C_90_H_152_O_76_	GF_14_	>100
8^b^ ^,^ ^d^	323.0978	8.89	C_12_H_20_O_10_	Difructose anhydride, isomer	>100	23^a^	1,304.4215^e^	15.70	C_96_H_162_O_81_	GF_15_	>100
9^b^ ^,^ ^c^	341.1091	9.43	C_12_H_22_O_11_	D (+)-sucrose	2.2	24^a^	1,385.4441	15.90	C_102_H_172_O_86_	GF_16_	>100
10^a^ ^,^ ^c^	503.1605	10.70	C_18_H_32_O_16_	1-Kestose	4.8	25^a^	1,466.4720^e^	16.01	C_108_H_182_O_91_	GF_17_	>100
11^a^ ^,^ ^c^	665.2148	11.47	C_24_H_42_O_21_	GF_3_	5.3	26^a^	1,547.4960^e^	16.32	C_114_H_192_O_96_	GF_18_	>100
12^a^ ^,^ ^c^	827.2650	12.09	C_30_H_52_O_26_	GF_4_	7.5	27^a^	1,628.5131^e^	16.51	C_120_H_202_O_101_	GF_19_	>100
13^a^ ^,^ ^c^	989.3250	12.61	C_36_H_62_O_31_	GF_5_	8.7	28^a^	1,709.5289^e^	16.72	C_126_H_212_O_106_	GF_20_	>100
14^a^ ^,^ ^c^	1,151.3725	13.09	C_42_H_72_O_36_	GF_6_	10.1	29^a^	1,790.5447^e^	16.96	C_132_H_222_O_111_	GF_21_	>100
15^a^ ^,^ ^c^	1,313.4269	13.50	C_48_H_82_O_41_	GF_7_	8.5						

a Note. The content of compounds in MOR was higher than that in PMOR.

b The content of compounds in PMOR was higher than that in MOR.

c Compared with a reference substance.

d Compound first discovered in PMOR.

e [M−2H]^2−^ value.

**TABLE 2:** Chemical components with larger difference in content between the samples of MOR and PMOR (Acquity BEH column).

No	*m/z*	RT/min	Formula	Compound	Factor of change	No	*m/z*	RT/min	Formula	Compound	Factor of change
1^a^	389.1085	1.05	C_16_H_22_O_11_	Monotropein isomer	1.5	14^b^	405.1029	1.81	C_16_H_22_O_12_	Shanzhiside methyl ester	3.1
2^a^ ^,^ ^c^	389.1080	1.33	C_16_H_22_O_11_	Monotropein	2.4	15^b^ ^,^ ^d^	713.2142	1.9	C_28_H_42_O_21_	Monotropein + glu + glu + glu	6.6
3^b^ ^,^ ^d^	941.2767	1.37	C_38_H_54_O_27_	Di-monotropein + glu	9.6	16^b^ ^,^ ^d^	695.2029	2.15	C_28_H_40_O_20_	Monotropein + glu + glu-H_2_O	88.3
4^a^ ^,^ ^c^	389.1077	1.39	C_16_H_22_O_11_	DDA	4.3	17^b^ ^,^ ^d^	761.2110	2.18	C_32_H_42_O_21_	Di-monotropein-H_2_O	88.3
5^b^ ^,^ ^d^	1103.3306	1.42	C_44_H_64_O_32_	Di-DAA + glu + glu	4.4	18^b^	1,097.3739	2.69	C_43_H_70_O_32_	Iridoid glycoside	264.5
6^b^ ^,^ ^d^	779.2241	1.67	C_32_H_44_O_22_	Di-DAA	3.4	19^b^	371.0972	2.86	C_16_H_20_O_10_	Unknown compound	11.9
7^b^ ^,^ ^d^	761.2179	1.69	C_32_H_42_O_21_	Di-DAA-H_2_O	5.3	20^b^	405.1396	2.90	C_17_H_26_O_11_	7-Hydroxy-deacetylcholoxalate	2.5
8^b^ ^,^ ^d^	389.1084	1.71	C_16_H_22_O_11_	DAA, isomer	220.4	21^b^ ^,^ ^d^	1,139.3855	4.15	C_45_H_72_O_33_	Iridoid glycoside	11.9
9^b^ ^,^ ^d^	551.1615	1.73	C_22_H_32_O_16_	DAA + glu	19.3	22^b^	1,417.4871	4.80	C_56_H_90_O_41_	Iridoid glycoside	274.6
10^b^ ^,^ ^d^	875.2707	1.73	C_34_H_52_O_26_	DAA-Glu (Fru)-Glu (Fru)-Glu (Fru)-Glu (Fru)	29.2	23^b^	463.2147	5.09	C_21_H_36_O_11_	Iridoid glycoside	8.3
11^b^	371.0972	1.75	C_16_H_20_O_10_	DAA-H_2_O	3.5	24^a^	593.1520	6.83	C_27_H_30_O_15_	1,3-Dihydroxy-2-methylanthraquinone-3-O-*β*-D-fructofuranose-(l→2)-*β*-D- fructofuranoside	1.5
12^b^	551.1616	1.78	C_22_H_32_O_16_	DAA + glu	48.2	25^a^	593.1520	7.42	C_27_H_30_O_15_	1,3-Dihydroxy-2-methylanthraquinone-3-O-*β*-fructofuranose-(l→2)-*β*-D- fructofuranoside, isomer	68.9
13^a^	405.1035	1.78	C_16_H_22_O_12_	Shanzhiside methyl ester or its isomers	3.0	26^a^	563.1413	7.49	C_26_H_28_O_14_	1-Hydroxy-anthraquinone-3-O-*β*-D-glucopyranoside-(1→6)-*β*-D-glucopyranoside	10.9

a Note. The content of compounds in MOR was higher than that in PMOR.

b The content of compounds in PMOR was higher than that in MOR.

c Compared with a reference substance.

d Compound first discovered in PMOR.

### 3.3 Identification of the Main Different Components

#### 3.3.1 Identification of Sugars

**FIGURE 3:**
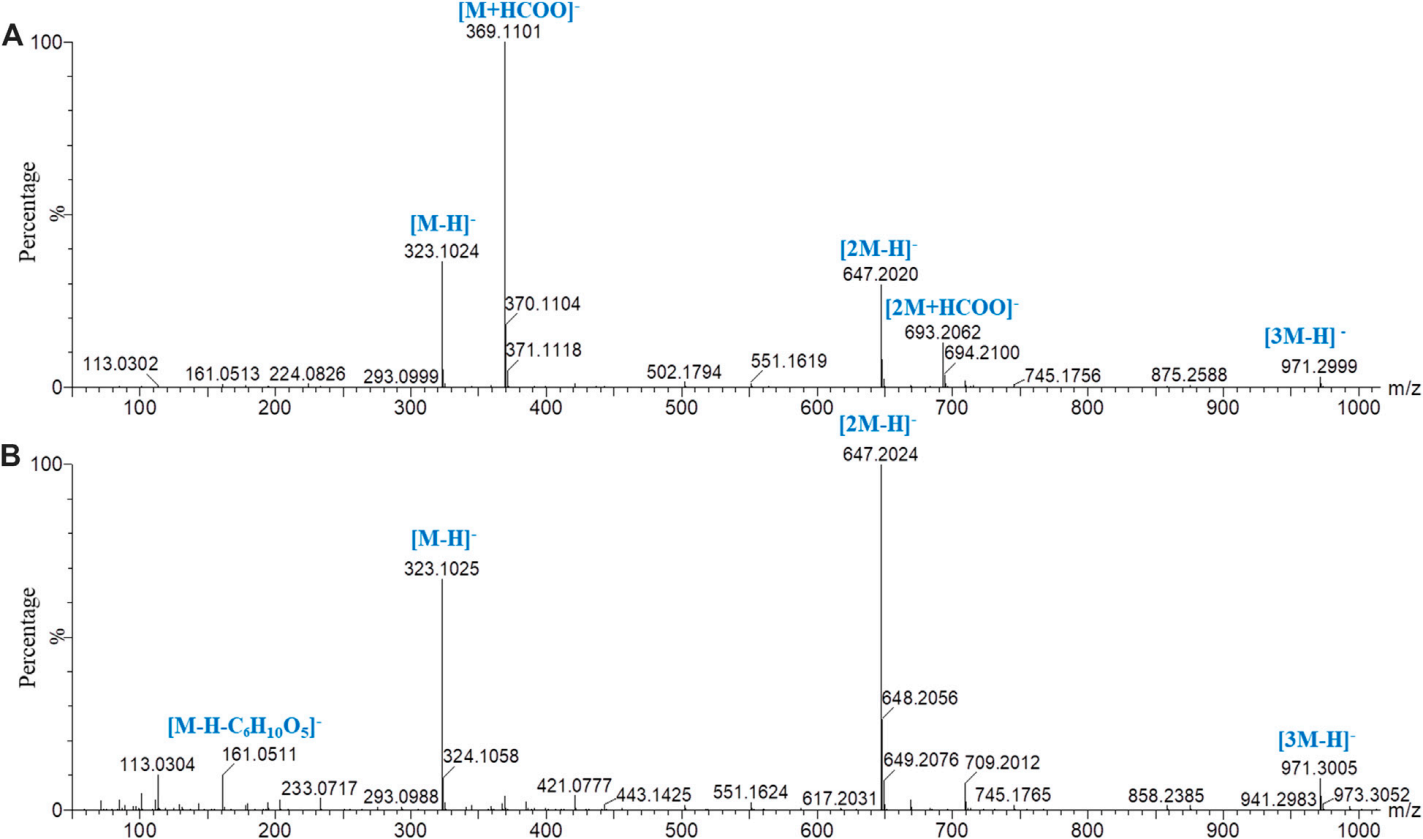
Mass spectrometry of peak *3* in PMOR in negative ion mode with chromatographic condition 2. **(A)** Low-energy mass spectrometry; **(B)** high-energy mass spectrometry.

By comparing the retention time and fragment ion information with those of the reference substances, the structures of 31 sugars, including difructose anhydrides (DFAs), sucrose, GF2–GF21, and isomers of GF2–GF5, were determined (Supplementary Table S1). Peak *3* in Figure 1A is an example to educate about the fragmentation pathway of DFAs. In the low-energy negative ion mass spectrum (Figure 3A), the ions of *m/z* 323.1024 [M‒H]^−^, 369.1101 [M+HCOO]^−^, 647.2020 [2M‒H]^−^, 693.2062 [2M+HCOO]^−^, and 971.2999 [3M‒H]^−^ were observed, indicating that the molecular formula was C_12_H_20_O_10_. In the high-energy mass spectrum (Figure 3B), the fragment ions of *m/z* 179.0615, 161.0511, and 113.0304 were found, suggesting the structure of a DFA compound. Similarly, the other six DFA compounds could be identified separately, and the results are shown in Supplementary Table S1.

**FIGURE 4:**
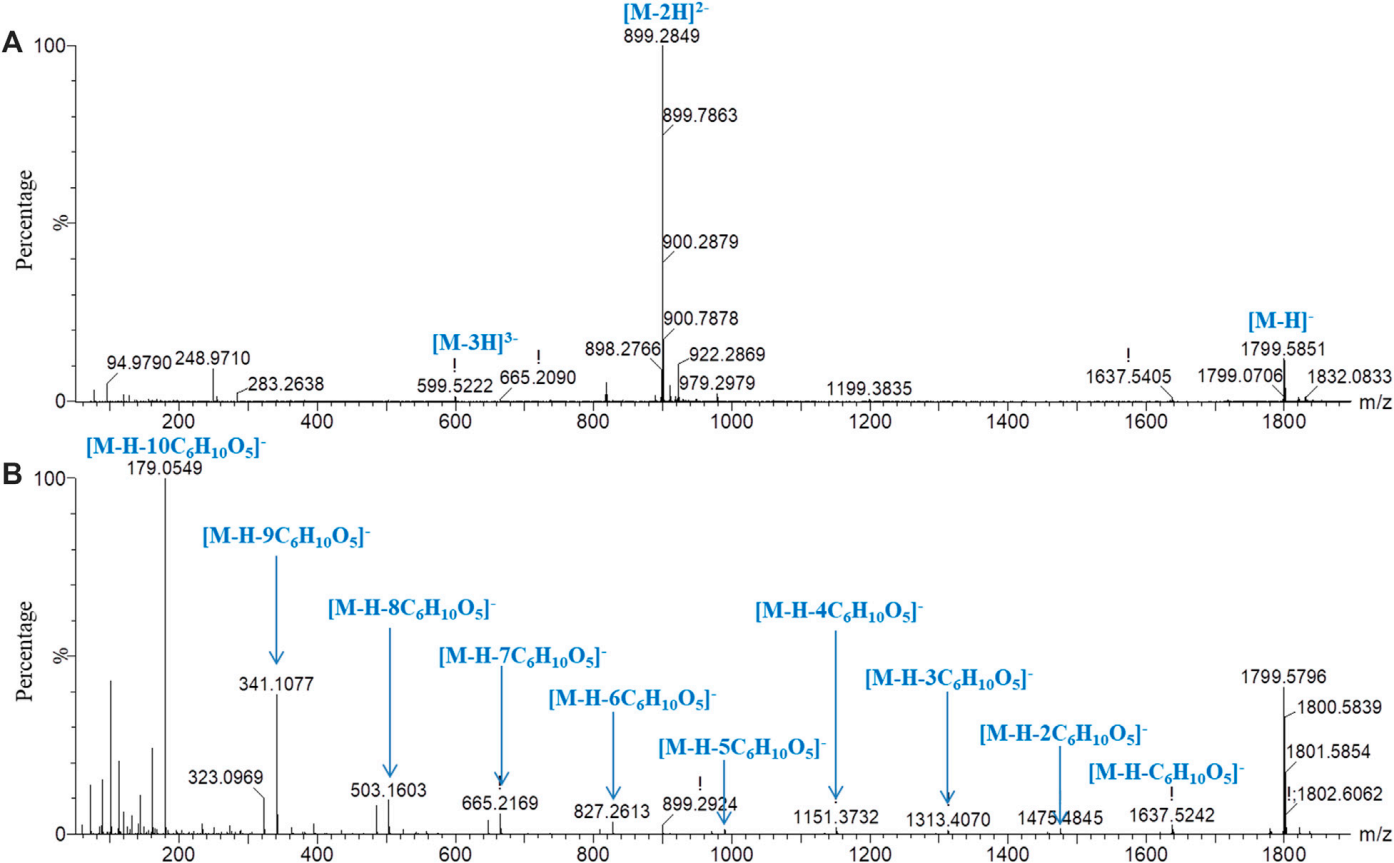
Mass spectrometry of peak *23* in negative ion mode with chromatographic condition 2. **(A)** Low-energy mass spectrometry; **(B)** high-energy mass spectrometry.

For oligosaccharides with molecular mass greater than 2,500 Da, although the [M‒H]^‒^ and characteristic fragment *m/z* greater than 2,500 Da could not be accurately detected, the characteristic fragment ions of [M‒2H]^2−^ and [M‒3H]^3−^ could be accurately detected in low-energy mass spectrometry for the reference substance of GFn (*n* ˃ 5). Therefore, the oligosaccharides with molecular mass greater than 2,500 Da could be detected and identified. With peak *23* as an example, the fragmentation pathway was studied. In the low-energy negative ion mass spectrum (Figure 4A), the ions of *m/z* 1,799.5851 [M‒H]^‒^, 899.2849 [M‒2H]^2‒^, and 599.2222 [M‒3H]^3‒^ were observed, indicating a molecular formula of C_66_H_112_O_56_. In the high-energy mass spectrum (Figure 4B), a series of fragment ions sequentially lost the fragment of C_6_H_10_O_5_, suggesting that the compound was composed of 11 hexoses (GF_10_).

Similarly, the structures of the other sugars could be identified, and the results are shown in Supplementary Table S1.

The results revealed that the contents of GFn (*n* = 2–21) were higher in MOR than in PMOR, whereas the contents of DFAs and sucrose were higher in PMOR than in MOR. DFAs have a variety of physiological functions, such as enhancing immunity and diuresis and promoting calcium absorption (Mellet and García Fernández, 2010). DFAs are the main components of medicines and certain functional foods (Cheng et al., 2021). The effect of invigorating kidneys and tonifying yang of PMOR may be related to the increased DFA content. Because of the different physiological activities of DFAs of different configurations, further research is needed to determine the exact configurations of DFAs in PMOR.

#### 3.3.2 Identification of Iridoids

**FIGURE 5:**
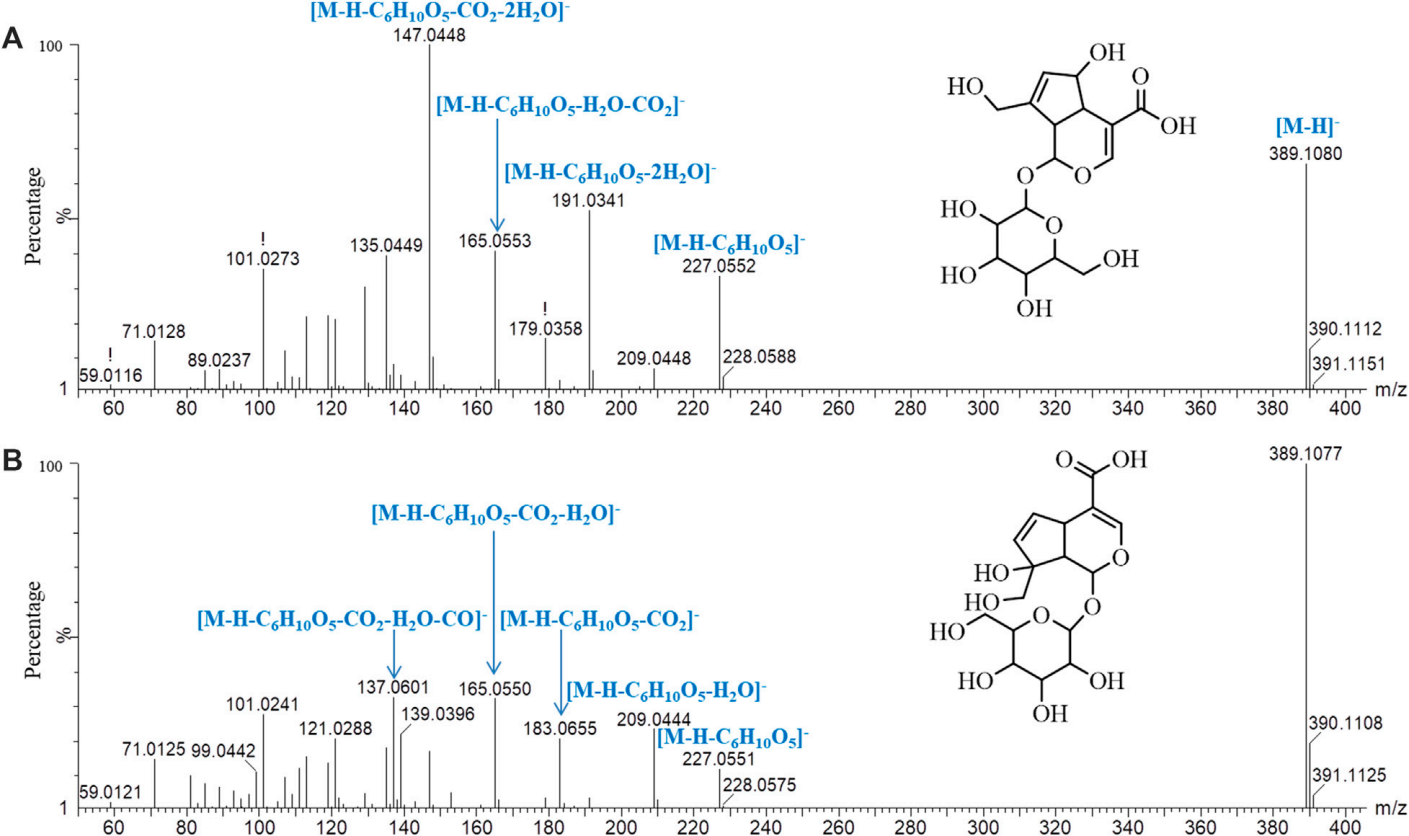
Mass spectrograms and major cleavage pathways of monotropein **(A)** and DDA **(B)** at high energy in negative ion mode.

**FIGURE 6:**
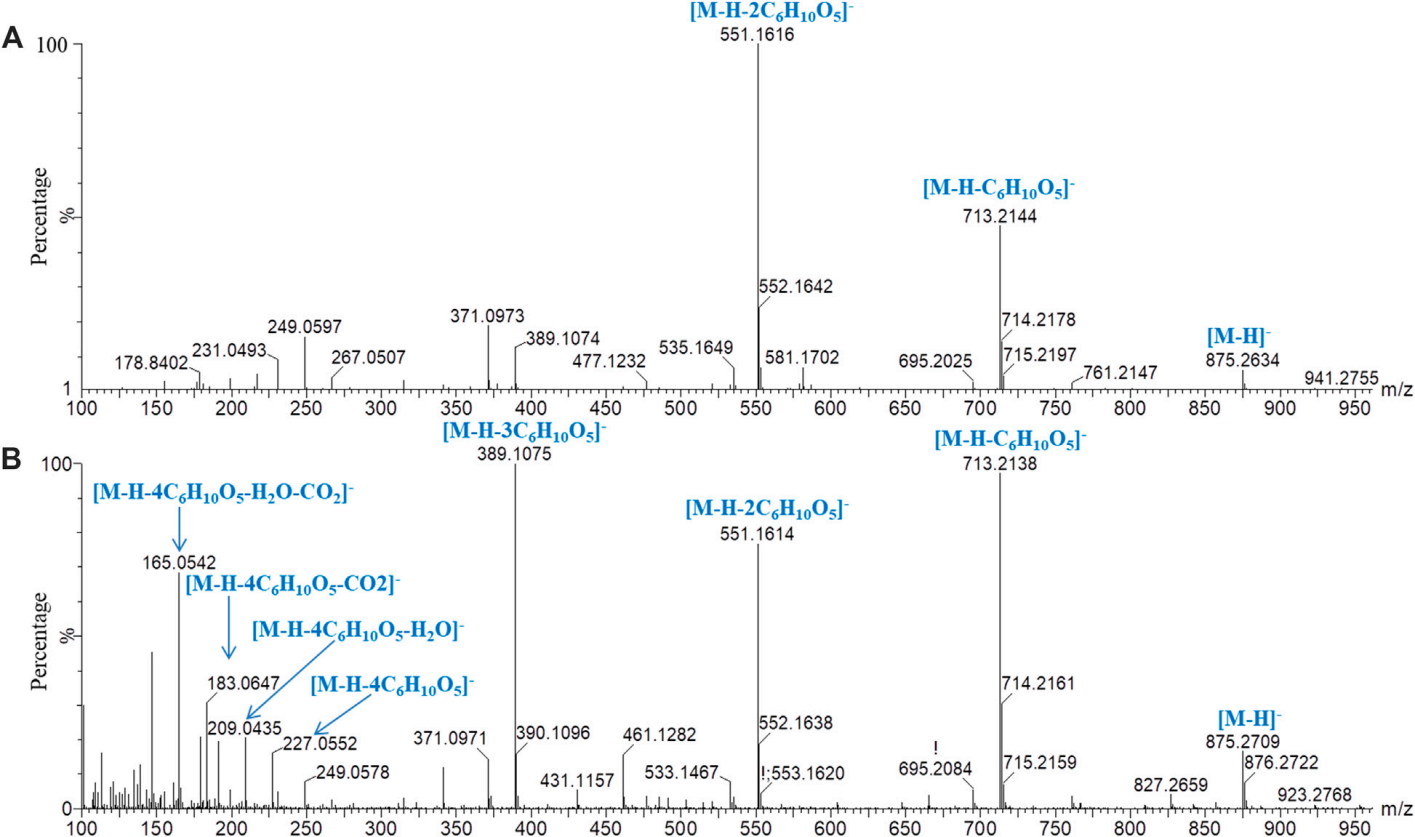
Mass spectrograms and major cleavage pathways of peak *47* (*m/z* 875.2664) in negative ion mode. **(A)** Low-energy mass spectrometry; **(B)** high-energy mass spectrometry.

Iridoids and their glycosides are the main terpenoids with a cyclopentenoid structure in MOR, and their mass fragment ions are mostly produced after the parent ion has lost its substituents (Li et al., 2008; Wu et al., 2013). Monotropein and DAA are the main iridoid glycosides in MOR. They are isomers that differ structurally in the position of the cyclopentene double bond and the positions of the −OH substituents with the formula C_16_H_22_O_11_. It was effective for separating monotropein and DAA in MOR and PMOR with an Acquity BEH column (Figures 1C,D). In the mass spectrum in low-energy negative, monotropein and DAA showed the quasimolecular ion peaks of *m/z* 389.1080 [M‒H]^−^, 425.0844 [M+Cl]^−^, 435.1136 [M+HCOO]^−^, and 779.2240 [2M‒H]^−^. In the mass spectrum in high-energy negative ion, a series of fragment ions of *m/z* 227.0552, 209.0448, 191.0341, 183.0655, 165.0533, 147.0448, 137.0601, and 135.0449 were found indicating that the molecular ion lost a glycosyl group, H_2_O, CO_2_, and CO, and glucose, and its fragment ions of *m/z* 179.0358, 161.0443, 101.0273, and 71.0128 were also observed (Figure 5). Although the fragment ions of the two compounds were almost identical, their abundance ratios of *m/z* 227.0552, 209.0448, 191.0341, 183.0655, and 165.0553 were significantly different. In monotropein, the abundance ratio of these five ions was 1:0.2:1.5:0.1:0.8, the signal of *m/z* 191.0341 was strong, and the signals of *m/z* 209.0448 and 183.0636 were weak. In DAA, the abundance ratio of the five ions was 1:1.8:0.1:1.5:2.2, the signal of *m/z* 191.0341 was weak, and the signals of *m/z* 209.0444 and 183.0655 were strong. The results of the two compounds were just the opposite. According to the characteristics of the abundance ratios of these ions, the corresponding derivatives of monotropein and DAA could be identified. Take peak *47* as an example to analyze the structure identification process. In the high-energy negative ion mode, *m/z* 875.2709, 713.2138, 551.1614, 389.1075, and 227.0552 corresponded to the [M − H]^−^ ion and the ions after the losses of four consecutive C_6_H_10_O_5_ groups, respectively; *m/z* 227.0552, 209.0435, 191.0318, 183.0647, and 165.0542 were characteristic fragment ions of iridoids, and the abundance ratio was 1:1.4:0.1:1.5:2.2 (Figure 6). The compound was determined to be the derivative of DAA and identified as DAA-Glu (Fru)-Glu (Fru)-Glu (Fru)-Glu (Fru), which was a new compound in PMOR. In the same way, according to the fragment ions and their abundance ratio under high energy, peaks *40*, *47*, *52*, *54*, and *55* were determined to be the derivatives of monotropein; peaks *42*, *43*, *44*, *45*, *46*, *48*, and *49* were the derivatives of DAA. The identification results for iridoid components are listed in Supplementary Table S2. Results showed that the contents of iridoids, including monotropein and DAA, were higher in MOR than in PMOR; the content of iridoid glycoside derivatives was higher in PMOR than in MOR.

The derivatives of monotropein and DAA were both potential new compounds that were first discovered in PMOR. The generation of these new iridoid glycosides may be related to the addition reaction of iridoid and sugar during the steaming process; in the process of steaming MOR, oligosaccharides are degraded to produce a large amount of glucose, fructose, and sucrose, and these sugars could promote the occurrence of iridoid glycosylation; or during the steaming process, the activity of certain enzymes in MOR changes, resulting in the combination of iridoid glycosides and sugars stored in inactive forms in MOR to produce new iridoid glycosides (Li et al., 2021). Further research on the production mechanism of new iridoid glycosides in PMOR is needed.

#### 3.3.3 Identification of Anthraquinones

**FIGURE 7:**
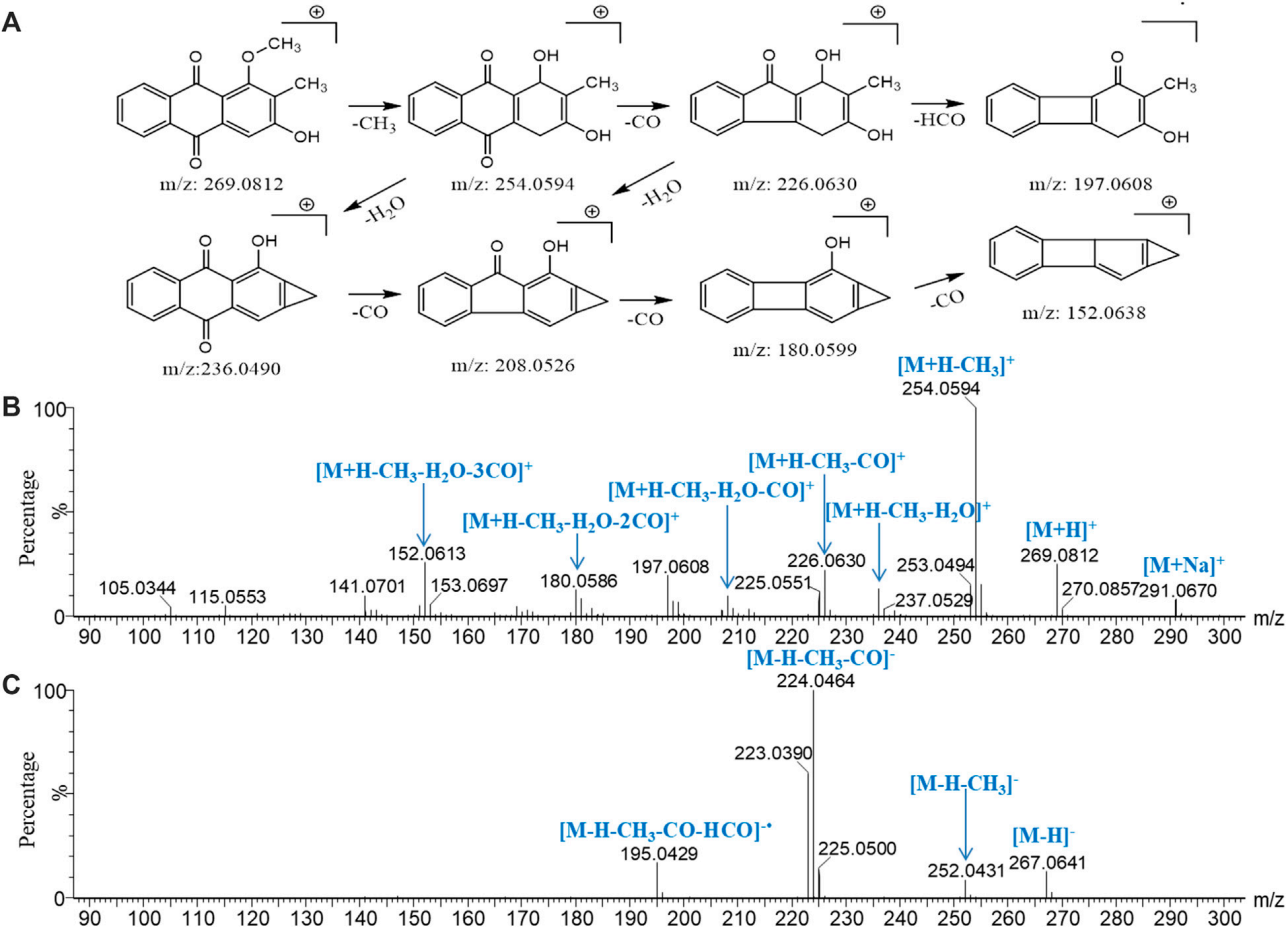
Major cleavage pathway **(A)** and mass spectrogram of rubiadin-1-methyl ether at high energy in positive ion mode **(B)** and in negative ion mode **(C)**.

Because of the conjugated structure of the anthraquinone compound, the main fragmentation mode is to gradually lose CO and side-chain substituents (‒OH, ‒CH_3_, ‒OCH_3_, and ‒COOH) in the core group and retain the conjugated structure (Wang et al., 1992). With peak *96* (rubiadin-1-methyl ether) as an example, the fragmentation pathway of anthraquinone was analyzed (Figure 7). In the low-energy mass spectrum of positive ions, *m/z* 269.0812 [M+H]^+^ and 291.0635 [M+Na]^+^ ions were detected. The high-energy mass spectrum showed fragment ions of *m/z* 254.0594 [M+H‒CH_3_]^+^, 236.0490 [M+H‒CH_3_‒H_2_O]^+^, 226.0630 [M+H‒CH_3_‒CO]^+^, 208.0526 [M+H‒CH_3_‒CO‒H_2_O]^+^, 180.0599 [M+H‒CH_3_‒H_2_O-2CO]^+^, and 152.0638 [M+H‒CH_3_‒H_2_O‒3CO]^+^; *m/z* 197.0608 was the free radical ion formed after [M+H‒CH_3_‒CO]^+•^ lost HCO^•^. In the negative ion mode, only the ion of *m/z* 267.0675 [M‒H]^−^ was detected in the low-energy mass spectrum; the ions of *m/z* 252.0418 [M‒H‒CH_3_]^−^, 224.0391 [M‒H‒CH_3_‒CO]^−^, and 195.0429 [M‒H‒CH_3_‒CO‒HCO]^−•^ were detected in the high-energy mass spectrum. According to the accurate molecular weight, fragmentation pattern, and characteristic fragments, peaks *77*, *79*, *80*, *85*, *86*, *87*, *88*, *89*, *91*, *92*, *93*, *94*, *95*, *96*, *98*, *99*, *100*, and *102* were determined to be anthraquinone compounds; the identification results are shown in Supplementary Table S2.

The contents of 1,3-dihydroxy-2-methylanthraquinone-3-*O*-*β*-D-fructofuranose-(l→2)-*β*-D- fructofuranoside and 1-hydroxy-anthraquinone-3-*O*-*β*-D-fructofuranose-(1→6)-*β*-D- glucopyranoside (Wang et al., 1992; Wang et al., 2019) were higher in MOR than in PMOR, possibly because the glycoside chain of the anthraquinone glycoside is broken during the steaming process, which causes the relative content of the anthraquinone glycoside to decrease in PMOR.

## 4 Conclusion

In this study, a method was developed to systematically study the similarities and differences in the overall chemical composition of MOR and PMOR. The structures of 110 compounds were identified, and 55 components were significantly different in content between MOR and PMOR. A total of 29 compounds showed higher contents in MOR than in PMOR, including GF_2_ to GF_21_, 1,3-dihydroxy-2-methylanthraquinone-3-*O*-*β*-D-fructofuranose-(l→2)-*β*-D-fructofuranoside and iridoid glycosides, such as monotropein, and DAA. A total of 26 compounds showed higher content in PMOR than in MOR, including DFAs, sucrose, and iridoid glycoside derivatives. DFAs and iridoid glycoside derivatives were found in PMOR for the first time. The changes in its efficacy may be closely related to the substantial changes in these components after MOR is processed by steaming. DFAs have a variety of physiological functions and have received extensive attention in the food and pharmaceutical industries. DFAs and iridoid glycoside derivatives should be purified and identified in a further study, and their activities should be determined. The results would provide a scientific basis for research on the therapeutic material basis of MOR and PMOR, and provide an effective method for the comparison of other processed and nonprocessed Chinese medicines.

## Data Availability

The original contributions presented in the study are included in the article/Supplementary Material, further inquiries can be directed to the corresponding authors.
