# Supplementary material for: Structural Characterization and Discrimination of Morinda officinalis and Processing Morinda officinalis Based on Metabolite Profiling Analysis
Source: Front Chem. 2022 Jan 21;9:803550. doi: 10.3389/fchem.2021.803550 (PMC8815813; doi:10.3389/fchem.2021.803550)
Supplement: Supplementary file 1 [file DataSheet1.docx]

Supporting Information

Table 1S HR-ESI-MS data of the identification compounds from MOR and PMOR by UPLC-Q-TOF MS (Acquity BEH amide column)

| PeakNo. | RT   (min) | Neutral  mass (Da) | Mass  *m/z* | Formula | Mass error (ppm) | Adducts | Fragments | Compound name |
| --- | --- | --- | --- | --- | --- | --- | --- | --- |
| **1^a,c^** | 5.74 | 390.1162 | 389.1085 | C_16_H_22_O_11_ | -1.0 | -H | 389.1077, 227.0551, 209.0444, 191.0318, 183.0655, 165.0550, 147.0424, 137.0601, 101.0241 | Deacetylasperulosidic acid |
| **2^a,c^** | 5.84 | 390.1162 | 389.1085 | C_16_H_22_O_11_ | -1.0 | -H | 389.1080, 227.0552, 209.0448, 191.0341, 183.0636, 165.0533, 147.0448, 137.0583, 101.0273 | Monotropein |
| **3^b,d^** | 7.03 | 324.1056 | 323.1024 | C_12_H_20_O_10_ | -0.37 | -H | 323.1024, 179.0642, 161.0511, 113.00304, 101.0285 | Difructose anhydride, DFAs |
| **4^b,d^** | 7.25 | 324.1056 | 323.0981 | C_12_H_20_O_10_ | -1.2 | -H | 323.0981, 161.0437, 101.0228 | Difructose anhydride, DFAs isomer |
| **5^b,d^** | 7.55 | 324.1056 | 323.0979 | C_12_H_20_O_10_ | 0.3 | -H | 323.0979, 179.0568, 161.0452, 113.0245, 101.0210, 85.0291, 71.0135 | Difructose anhydride, DFAs isomer |
| **6^b,d^** | 7.93 | 324.1056 | 323.0987 | C_12_H_20_O_10_ | 0.3 | -H | 323.0987, 179.0568, 161.0452, 113.0245, 101.0210, 85.0291, 71.0135 | Difructose anhydride, DFAs isomer |
| **7^b,d^** | 8.25 | 324.1056 | 323.0980 | C_12_H_20_O_10_ | 0.6 | -H | 323.0980, 179.0564, 161.0450, 113.0242, 101.0240, 71.0130 | Difructose anhydride, DFAs isomer |
| **8^b,d^** | 8.89 | 324.1056 | 323.0978 | C_12_H_20_O_10_ | -0.6 | -H | 323.0978, 179.0563, 161.0454, 101.0238 | Difructose anhydride, DFAs isomer |
| **9** | 9.07 | 486.1585 | 485.1504 | C_18_H_30_O_15_ | -0.4 | -H | 485.1519, 341.1087, 323.0974, 179.0561, 161.0464, 101.0236 | α-D-Glucopyranosyl-(1→4)-2,3-anhydro-α-D-mannopyranosyl-(1→4)-D-glucopyranose |
| **10^b,c^** | 9.43 | 342.1162 | 341.1091 | C_12_H_22_O_11_ | 2.1 | -H | 341.1091, 179.0563, 161.0466, 101.0244, 71.0128 | D(+)-Sucrose |
| **11** | 10.47 | 504.1690 | 503.1615 | C_18_H_32_O_16_ | 0.6 | -H | 503.1615, 341.11079, 179.0550, 161.0444, 101.0231 | 1-KESTOSE isomer |
| **12^a,c^** | 10.70 | 504.1690 | 503.1605 | C_18_H_32_O_16_ | -1.4 | -H | 503.1605, 341.1078, 323.0970, 179.0547, 161.0445, 101.0230 | 1-KESTOSE |
| **13** | 11.28 | 666.2201 | 665.2147 | C_24_H_42_O_21_ | 1.1 | -H | 665.2147, 503.1599, 341.1075, 179.0548, 161.0441, 101.0231 | GF_3_ isomer |
| **14^a,c^** | 11.46 | 666.2201 | 665.2148 | C_24_H_42_O_21_ | 1.2 | -H | 665.2148, 503.1620, 485.1512, 341.1081, 323.0975, 179.0550, 161.0513, 101.0232 | GF_3_ |
| **15** | 11.93 | 828.2748 | 827.2683 | C_30_H_52_O_26_ | 1.7 | +HCOO^-^ | 827.2683, 665.2147, 503.1615, 341.11082, 179.0556, 161.0447,101.0233 | GF_4_ isomer |
| **16^a,c^** | 12.09 | 828.2748 | 827.2650 | C_30_H_52_O_26_ | -2.3 | +HCOO^-^ | 827.2650, 665.2120, 503.1596, 485.1496, 413.1281**, 341.1075, 323.0966, 179.0545, 161.0443, 101.0232 | GF_4_ |
| **17** | 12.45 | 990.3275 | 989.3176 | C_36_H_62_O_31_ | -2.1 | -H | 989.3176, 827.2708, 665.2146, 503.1606, 413.1281**, 341.1089, 179.0570, 101.0242 | GF_5_ isomer |
| **18^a,c^** | 12.61 | 990.3275 | 989.3250 | C_36_H_62_O_31_ | -3.9 | -H | 989.3250, 827.2717, 485.1504**, 665.2158, 341.1066, 323.0970, 179.0544, 161.0455, 113.0243, 101.0243 | GF_5_ |
| **19^a,c^** | 13.09 | 1152.3803 | 1151.3725 | C_42_H_72_O_36_ | 1.6 | -H | 1151.3725, 989.3195, 827.2662, 665.2139, 575.1819**, 503.1618, 341.1081, 323.0977, 179.0553, 161.0449, 101.0236 | GF_6_ |
| **20^a,c^** | 13.50 | 1314.4331 | 1313.4269 | C_48_H_82_O_41_ | 1.2 | -H,-2H | 1313.4269, 1133.3699, 989.3141, 827.2678, 656.2136, 647.2029**, 503.1621, 485.1516, 341.1083, 179.0551, 161.0444, 101.0233 | GF_7_ |
| **21^a,c^** | 13.87 | 1476.4860 | 1475.4782 | C_54_H_92_O_46_ | -1.4 | -H, -2H | 1475.4782, 1313.3575, 989.3329, 827.2573, 737.2355**, 665.2105, 503.1595, 341.1069, 323.0970, 179.0548, 161.0444, 101.0229 | GF_8_ |
| **22a,c** | 14.21 | 1638.5388 | 1637.5297 | C_60_H_102_O_51_ | -0.8 | -H,-2H | 1637.5297, 818.2612**, 665.2141, 503.1619, 341.1074, 323.0968, 179.0549, 161.0443, 101.0232 | GF_9_ |
| **23^a,c^** | 14.52 | 1800.5916 | 1799.5851 | C_66_H_112_O_56_ | 1.2 | -H, -2H | 1799.5796, 1637.5242, 1475.4845, 1313.4070, 1151.3732, 899.2924**, 827.2613, 665.2169, 599.5222***, 503.11603, 341.1077, 179.0549, 161.0454, 101.0241 | GF_10_ |
| **24^a,c^** | 14.80 | 1962.6444 | 1961.6327 | C_72_H_122_O_61_ | -2.0 | -H,-2H | 1961.6327, 980.3115**, 827.2578, 665.2150, 503.1605, 341.1077, 179.0550, 161.0444, 101.0232 | GF_11_ |
| **25^a^** | 15.05 | 2124.6973 | 2123.6873 | C_78_H_132_O_66_ | -1.0 | -H,-2H | 2123.6873, 1061.3384**, 827.2621, 665.2122, 503.1591, 341.1067, 179.0551, 161.0448, 101.0236 | GF_12_ |
| **26^a^** | 15.30 | 2286.7501 | 2285.7463 | C_84_H_142_O_71_ | 1.7 | -H,-2H | 22857463, 1142.3676**, 989.3185, 827.2653, 665.2132, 503.1606, 341.1079, 179.0554, 101.0237 | GF_13_ |
| **27^a^** | 15.52 | 2448.8029 | 2447.7971 | C_90_H_152_O_76_ | 0.1 | -H,-2H | 2447.7971, 1223.8965**, 989.3237, 827.2673, 665.2119, 503.1606, 341.1090, 179.0555, 161.0456 | GF_14_ |
| **28^a^** | 15.70 | 1304.4200* | 1304.4215** | C_96_H_162_O_81_ | -0.1 | -2H | 1304.4215, 1151.3821, 989.3322, 827.2650, 665.2147, 503.1583, 341.1083, 179.0553, 101.0238 | GF_15_ |
| **29^a^** | 15.90 | 1385.4465* | 1385.4441 | C_102_H_172_O_86_ | -0.2 | -2H | 1385.4441, 989.3267, 827.2621, 665.2233, 503.1517, 341.1070, 179.0551, 101.0234 | GF_16_ |
| **30^a^** | 16.01 | 1466.4729 | 1466.4720** | C_108_H_182_O_91_ | -0.1 | -2H, -3H | 1466.4720, 1151.3740, 977.3007***，989.3041, 827.2637, 665.2009, 341.1073, 179.0554, 161.0439, 101.0232 | GF_17_ |
| **31^a^** | 16.32 | 1547.4993* | 1547.4960** | C_114_H_192_O_96_ | -2.1 | -2H, -3H | 1547.4960, 1031.6537***, 827.2684, 665.2084, 341.1097, 179.0551 | GF_18_ |
| **32^a^** | 16.51 | 1628.5257* | 1628.5131** | C_120_H_202_O_101_ | -2.3 | -2H, -3H | 1628.5135, 1085.3356***, 989.3079, 827.2599, 503.1584, 341.1128, 179.0553 | GF_19_ |
| **33^a^** | 16.72 | 1709.5521* | 1709.5289** | C_126_H_212_O_106_ | -4.6 | -2H, -3H | 1709.5289, 1139.3539***, 989.3082,827.2549, 665.2134, 503.1641 | GF_20_ |
| **34^a^** | 16.96 | 1790.5785* | 1790.5447 | C_132_H_222_O_111_ | -4.5 | -2H, -3H | 1790.5447, 1193.7029***, 989.3080, 827.2541, 665.2115 | GF_21_ |

Note: ^a^ the content of compounds in MOR was higher than that in PMOR; ^b^ the content of compounds in PMOR was higher than that in MOR; c compare with a reference substance; ^d^ the compound was first discovered in PMOR. * [M-2H]^2-^ , theoretical value; ** [M-2H]^2-^ detection value; *** [M-3H]^3-^ detection value.

Table 2S HR-ESI-MS data of the identification compounds from MOR and PMOR by UPLC-Q-TOF MS (Acquity BEH column)

| Peak  No. | RT  (min) | Neutral  mass (Da) | Mass *m/z* | Formula | Mass error  (ppm) | Adducts | Fragments | Compound name |
| --- | --- | --- | --- | --- | --- | --- | --- | --- |
| **35** | 0.55 | 196.0583 | 195.0495 | C_6_H_12_O_7_ | -4.6 | -H | 195.0495, 179.0540, 161.0445, 101.0229 | Gluconic acid |
| **36** | 0.70 | 134.0215 | 133.0139 | C_4_H_6_O_5_ | -2.4 | -H | 133.0139, 89.3224 | hydroxy-butanedioic acid |
| **37^a^** | 1.05 | 390.1158 | 389.1085 | C_16_H_22_O_11_ | 0.0 | -H | 389.1085, 227.0548, 209.0438, 191.0318, 179.0321, 165.0532, 147.0424, 101.0223 | Monotropein isomer |
| **38** | 1.25 | 118.0266 | 117.019 | C_4_H_6_O_4_ | -2.5 | -H | 117.0190 | Succinic acid |
| **39 ^a,c^** | 1.33 | 390.1158 | 389.1085 | C_16_H_22_O_11_ | 0.0 | -H | 389.1080, 227.0552, 209.0448, 191.0318, 179.0358, 165.0553, 147.0448, 101.0273 | Monotropein |
| **40^b,d^** | 1.37 | 942.2852 | 941.2767 | C_38_H_54_O_27_ | -0.4 | -H | 941.2767, 779.2228, 389.1071, 227.0541, 209.0435, 191.0329, 179.0324, 165.0536, 147.0431, 101.0227 | Di-monotropein+glu |
| **41^a,c^** | 1.39 | 390.1162 | 389.1085 | C_16_H_22_O_11_ | -1.0 | -H ^-^ | 389.1077, 227.0551, 209.0444, 183.0655, 165.0550, 137.0601, 101.0241 | DDA |
| **42^b,d^** | 1.42 | 1104.338 | 1103.3306 | C_44_H_64_O_32_ | -0.6 | -H | 1103.3306, 941.2764, 779.2137, 551.1608, 389.1075, 227.0544, 183.0642, 165.0538, 137.0592, 113.0228, 101.0229 | Di- DAA+glu+glu |
| **43^b,d^** | 1.67 | 780.2314 | 779.2241 | C_32_H_44_O_22_ | -0.1 | -H | 779.2241, 551.1599, 389.1088, 227.0517, 209.0438, 183.0636, 165.0532, 137.0583, 101.0223 | Di-DAA |
| **44^b,d^** | 1.69 | 762.2219 | 761.2179 | C_32_H_42_O_21_ | -1.6 | -H | 761.2179, 551.1599, 389.1088, 227.0517, 209.0438, 183.0636, 165.0532, 137.0583, 101.0223 | Di- DAA-H_2_O |
| **45^b,d^** | 1.71 | 390.1162 | 389.1084 | C_16_H_22_O_11_ | -1.5 | -H | 389.1084, 227.0517, 209.0438, 183.0636, 165.0532, 137.0583, 101.0223 | DAA, isomer |
| **46^b,d^** | 1.73 | 552.1690 | 551.1615 | C_22_H_32_O_16_ | 0.5 | -H | 551.1615, 389.1082, 227.0517, 209.0438, 183.0636, 165.0532 | DAA+glu |
| **47^b,d^** | 1.73 | 876.2747 | 875.2709 | C_34_H_52_O_26_ | 0.0 | -H | 875.2709, 713.2138, 551.1614, 389.1075, 209.0435, 191.0341, 165.0542, 147.0429 | DAA-Glu(Fru)-Glu(Fru)-Glu(Fru)-Glu(Fru) |
| **48^b^** | 1.75 | 372.1048 | 371.0972 | C_16_H_20_O_10_ | -1.6 | -H | 371.0972, 323.0931, 227.0544, 209.0440, 191.0333, 183.0647, 165.0539, 147.0434, 137.0591, 101.0299 | DAA-H_2_O |
| **49^b^** | 1.78 | 552.1690 | 551.1616 | C_22_H_32_O_16_ | 0.7 | -H | 551.1616, 389.1078, 227.0544, 209.0437, 183.0647, 165.0539, 147.0437, 135.0437, 101.0229 | DAA+glu |
| **50^a^** | 1.78 | 406.1475 | 405.1035 | C_16_H_22_O_12_ | -0.8 | +HCOO^-^ | 405.1035, 361.1136, 343.1038, 199.0587, 181.0492, 179.0550, 101.0237 | shanzhiside methyl ester or its isomers |
| **51^b^** | 1.81 | 406.1103 | 405.1029 | C_16_H_22_O_12_ | -1.9 | +HCOO^-^ | 405.1029, 398.1078, 361.1145, 341.1082, 209.0440, 181.0489, 165.0540, 147.0436, 135.0435, 113.0232, 101.0237 | shanzhiside methyl ester |
| **52^b,d^** | 1.9 | 714.2219 | 713.2142 | C_28_H_42_O_21_ | -1.7 | -H | 713.2142, 551.1611, 389.1071, 341.1078, 227.0545, 209.0443, 191.0331, 179.0538, 165.0538, 147.0435, 101.0229 | Monotropein+glu+glu+glu |
| **53** | 1.98 | 346.1264 | 345.1185 | C_15_H_22_O_9_ | -1.6 | -H | 345.1185, 179.0529 | aucubin |
| **54^b,d^** | 2.15 | 696.2113 | 695.2029 | C_28_H_40_O_20_ | -3.2 | -H | 695.2029, 389.1034, 371.0966, 341.1046, 323.0969, 227.0554, 191.0332, 165.0539, 147.0429, 101.0231 | Monotropein+glu -H_2_O+glu |
| **55^b,d^** | 2.18 | 762.2219 | 761.2110 | C_32_H_42_O_21_ | -0.9 | -H | 761.2110, 389.1079, 371.0977, 227.0519, 191.0330, 179.0538, 165.0542, 147.0431, 101.0235 | Di-monotropein-H_2_O |
| **56^c^** | 2.42 | 404.1312 | 403.1239 | C_17_H_24_O_11_ | -1.7 | -H | 371.0974, 209.0447, 191.0333, 165.0544, 147.0438, 101.0236 | Gardenoside |
| **57^b^** | 2.69 | 1098.385 | 1097.3739 | C_43_H_70_O_32_ | -0.2 | -H | 1097.3739, 389.1061, 227.0544, 209.0446, 183.0641, 323.0950, 179.0543 | iridoid glycoside |
| **58** | 2.70 | 448.1217 | 447.1139 | C_18_H_24_O_13_ | 0.0 | -H | 447.1139, 341.1098, 179.0552, 139.0307 | 7-hydroxy asperulosidic acid |
| **59^b^** | 2.86 | 372.1048 | 371.0972 | C_16_H_20_O_10_ | -1.6 | -H | 371.0972 | unknown compound |
| **60^b^** | 2.90 | 406.1475 | 405.1396 | C_17_H_26_O_11_ | -0.2 | -H | 405.1396, 243.0852, 209.0, 445, 191.0327, 179.0539, 165.0536, 147.0435, 121.0276, 101.0227 | 7-hydroxy- deacetylcholoxalate |
| **61** | 3.17 | 404.1315 | 403.1240 | C_17_H_24_O_11_ | -0.2 | -H | 403.1240 | Geniposide |
| **62^c^** | 3.31 | 432.1268 | 431.1184 | C_18_H_24_O_12_ | -1.4 | -H | 431.1184, 251.0546, 225.0750, 179.0548, 165.0544, 147.0435, 121.0288, 113.0233, 101.0230 | asperulosidic acid |
| **63** | 3.53 | 294.1315 | 293.1236 | C_12_H_22_O_8_ | 0.0 | -H | 293.1236, 191.0529, 179.0553, 113.0235, 101.0238 | 1,5-Anhydro-2,6-dideoxy-3-O-α-D-glucopyranosyl-D-arabino-hexitol |
| **64** | 3.59 | 354.0951 | 353.0880 | C_16_H_18_O_9_ | 0.6 | -H | 353.0853 | Chlorogenic acid |
| **65** | 4.01 | 414.1162 | 413.1084 | C_18_H_22_O_11_ | -0.7 | -H, | 413.1084, 241.0483, 191.0338, 179.0516, 147.0428, 101.0223 | asperuloside |
| **66** | 4.11 | 364.1733 | 363.1651 | C_16_H_28_O_9_ | -1.1 | -H | 363.1651, 201.1112, 179.0578, 117.0181, 101.0239 | glycoside |
| **67^b,d^** | 4.15 | 1140.396 | 1139.3855 | C_45_H_72_O_33_ | -1.5 | -H | 1139.3855, 1007.3336, 861.2485, 363.1628, 341.1020, 227.1003, 209.0402, 179.0544, 161.0440, 147.0412, 113.0241, 101.0217 | iridoid glycoside |
| **68** | 4.27 | 496.2156 | 495.2092 | C_21_H_36_O_13_ | 2.8 | -H, | 495.2092, 341.1026, 179.0524, 117.0187, 113.0238, 101.0233 | unknown compounds |
| **69** | 4.42 | 388.1369 | 387.1291, | C_17_H_24_O_10_ | -2.3 | +HCOO^-^ | 387.1291, 161.0446, 113.0224, 101.0228 | geniposide |
| **70** | 4.70 | 520.1792 | 519.1647, | C_22_H_32_O_14_ | -2.1 | -H | 519.1647, 519.1703, 463.1675, 300.0984, | Geniposide+glu |
| **71^b^** | 4.80 | 1418.496 | 1417.4871 | C_56_H_90_O_41_ | -0.4 | -H | 1417.4871, 1285.4435, 708.2337, 513.1480, 227.0918, 209.0374, 191.0482, 179.0566, 165.0514, 161.0409, 113.0208, 101.0231 | iridoid glycoside |
| **72** | 5.01 | 464.2258 | 463.2163, | C_21_H_36_O_11_ | -3.5 | +HCOO^-^ | 463.2163, 331.1782, 227.0582, 209.0506, 193.0480, 179.0531, 161.0446, 147.0476, 113.0220, 101.0226 | iridoid glycoside |
| **73^b^** | 5.09 | 464.2258 | 463.2147, | C_21_H_36_O_11_ | -2.8 | -H | 463.2147, 227.0582, 179.0529, 161.0425, 113.0240, 101.0223 | iridoid glycoside |
| **74** | 5.25 | 404.1318 | 403.1227 | C_17_H_24_O_11_ | -4.7 | -H | 403.1227, 387.0907, 343.1016, 181.0473, 127.0375 | Geniposide |
| **75** | 5.31 | 536.1741 | 535.1635 | C_22_H_32_O_15_ | -0.4 | -H | 535.1635, 503.1661, 293.0861, 251.0788, | Leptosin |
| **76** | 5.96 | 192.0423 | 191.0343 | C_10_H_8_O_4_ | -2.1 | -H | 191.0343 | Scopoletin |
| **77^a^** | 6.83 | 594.1585 | 593.1520 | C_27_H_30_O_15_ | -1.1 | +HCOO | 593.1520, 341.1055, 227.0598 | 1, 3-dihydroxy-2-hydroxymethylanthraquinone-3-O-β-D- fructofuranose (l→2)-β-D- fructofuranoside |
| **78** | 7.23 | 188.1048 | 187.0961 | C_9_H_16_O_4_ | -3.7 | -H | 187.0961, 141.0932 | Azelaic Acid |
| **79^a^** | 7.42 | 594.1585 | 593.1520 | C_27_H_30_O_15_ | 1.4 | -H | 593.1520, 341.1055, 227.0598, 243.0823, 207.0294, 179.0552, 101.0217 | 1, 3-dihydroxy-2-methylanthraquinone-3-O-β-D-fructofuranose(l→2)-β-D- fructofuranoside, isomer |
| **80^a^** | 7.49 | 564.1479 | 563.1413 | C_26_H_28_O_14_ | 1.2 | -H | 563.1413, 269.0433, 251.0327, 237.0531 | 1-hydroxy-anthraquinone-3-O-β-D-glucopyranoside (1→6)-β-D-glucopyranoside |
| **81** | 7.66 | 478.2336 | 477.2322 | C_22_H_38_O_11_ | -2.9 | +HCOO | 477.2322, 315.1812, 161.0449, 101.0231 | (2,6,6-trimethyl-1-cyclohexen-1-yl)methyl 6-O-D-altropyranosyl-α-D-altropyranoside |
| **82** | 7.76 | 582.1585 | 581.1510 | C_26_H_30_O_15_ | -0.3 | -H | 581.1510, 256.0687 | asperuloside tetraacetate |
| **83** | 8.27 | 448.2301 | 447.2244 | C_21_H_36_O_10_ | -0.6 | -H | 447.2244, 315.1828, 179.0556, 161.0435, 101.2243, | 1-borneol-beta-apisyl-beta-glucopyranoside |
| **84^a^** | 8.48 | 448.2301 | 447.2220 | C_21_H_36_O_10_ | -0.6 | -H | 447.2220, 315.1792, 161.0455, 113.0221, 101.0222 | 1-borneol-beta-apisyl-beta-glucopyranoside |
| **85** | 9.49 | 254.0579 | 253.0499 | C_15_H_10_O_4_ | -2.9 | -H | 253.0499, 223.0390 | rubiadin isomer |
| **86** | 9.61 | 284.0683 | 283.0615 | C_14_H_8_O_4_ | 1 | -H | 283.0615, 253.0508, 239.0324, 10.0296, 195.0440 | physcion |
| **87** | 10.12 | 268.0372 | 267.0295 | C_15_H_8_O_5_ | -1.4 | -H | 267.0295, 223.0387, 195.0433 | 1-hydroxy-9,10-dioxo-9,10-dihydroanthracene-2-carboxylic acid |
| **88** | 10.34 | 268.0367 | 267.0294 | C_15_H_8_O_5_ | -1.8 | -H | 267.0294 | 1,3-dihydroxy-9,10-dioxo-9,10-dihydroanthracene-2-carbaldehyde |
| **89** | 10.98 | 270.0528 | 269.0453 | C_15_H_10_O_5_ | -1 | -H | 269.0453, 251.0337, 223.0390, 195.0441 | rhabarberone |
| **90** | 11.08 | 330.2406 | 329.2328 | C_14_H_8_O_5_ | -1.6 | -H | 329.2328, 229.1430, 211.1318, 139.1119 | tianshic acid |
| **91** | 11.14 | 330.2406 | 329.2319 | C_14_H_8_O_5_ | -4.5 | -H | 329.2319, 253.0485, 238.0255, 210.0306 | 1,2,4-trihydroxy-3,7-dimethoxy-6-methylanthracene-9,10-dione |
| **92^c^** | 11.16 | 254.0579 | 253.0489 | C_15_H_10_O_4_ | -6.7 | -H | 253.0498(29.08), 238.0253(25.12) | Chrysophanol |
| **93** | 11.65 | 284.0688 | 283.0610 | C_16_H_12_O_5_ | -0.6 | -H | 283.0610, 253.0122, 239.0329, 225.0194, 197.0218 | Physcion |
| **94** | 12.11 | 270.0528 | 269.0440 | C_15_H_10_O_5_ | -5.8 | -H | 269.0440, 254.0204, 226.0260 | 1,8-dihydroxy-3-methoxyanthracene-9,10-dione |
| **95** | 12.25 | 330.2406 | 329.2328 | C_14_H_8_O_5_ | -1.6 | -H | 329.2328, 211.1322, 171.1004, 139.11063 | Pseudopurpurin |
| **96** | 12.87 | 268.0736 | 267.0649 | C_16_H_12_O_4_ | -5.3 | -H | 267.0641, 252.0403, 224.0464 | rubiadin-1-methyl ether |
| **97** | 13.65 | 286.0477 | 285.0399 | C_15_H_10_O_6_ | -1.7 | -H | 285.0399, 270.0162, 242.0214, 214.0266 | Kaempferol |
| **98^c^** | 13.85 | 270.0528 | 269.0455 | C_15_H_10_O_5_ | -0.2 | -H | 269.0455, 254.0210, 237.0549 | 1,8-dihydroxy-3-(hydroxymethyl)anthracene-9,10-dione |
| **99^c^** | 14.84 | 284.0685 | 283.0608 | C_16_H_12_O_5_ | -1.3 | -H | 283.0608, 252.0401, 223.0381 | 1-hydroxy-2,3-dimethoxyanthracene-9,10-dione |
| **100** | 15.60 | 270.0528 | 269.0455 | C_15_H_10_O_5_ | -0.2 | -H | 269.0455, 254.0206, 237.0554 | 1,3-dihydroxy-2-methoxyanthracene-9,10-dione |
| **101** | 15.99 | 486.3345 | 485.3260 | C_30_H_46_O_5_ | -1.4 | -H | 485.3260, 467.3158 328.9845, 311.2201, 293.1793, | Rosamultic acid |
| **102^c^** | 16.07 | 254.0579 | 253.0499 | C_15_H_10_O_4_ | -2.9 | -H | 253.0499, 225.0537 | Rubiadin |
| **103** | 16.66 | 488.3502 | 487.3426 | C_30_H_48_O_5_ | 0.6 | -H | 487.3426, 469.3316, 443.3500, 427.3188, 405.3126 | rotundic acid |
| **104** | 16.75 | 488.3502 | 487.3418 | C_30_H_48_O_5_ | -0.8 | -H | 487.3418, | rotundic acid，isomer |
| **105** | 17.37 | 270.2559 | 315.2519 | C_17_H_34_O_2_ | -6.9 | +HCOO^-^ | 269.0428 | heptadecoic acid |
| **106** | 17.46 | 270.3451 | 269.2484 | C_18_H_34_O_2_ | -1.3 | -H | 269.2484, 255.2325, 251.2380, 223.2067 | Heptadecanoic acid |
| **107** | 17.63 | 264.2089 | 309.2054 | C_17_H_28_O_2_ | -5.6 | +HCOO^-^ | 309.2054 | r-linolenic acid |
| **108^a^** | 18.65 | 296.2351 | 295.2261 | C_18_H_32_O_3_ | -4.1 | -H | 295.2261 | Octanoic acid |
| **109** | 19.14 | 298.2508 | 297.2427 | C_18_H_34_O_3_ | -2.6 | -H | 297.2427, 279.2318 | ricinoleic acid |
| **110** | 19.61 | 294.2193 | 293.2112 | C_18_H_30_O_3_ | -1.7 | -H | 293.2112, 183.0122 | octadecadienoic acid |

Note: ^a^ the content of compounds in MOR was higher than that in PMOR; ^b^ the content of compounds in PMOR was higher than that in MOR; ^c,^ ompare with a reference substance; ^d^ compound first discovered in PMOR
